# Review: Radionuclide Molecular Imaging Targeting HER2 in Breast Cancer with a Focus on Molecular Probes into Clinical Trials and Small Peptides

**DOI:** 10.3390/molecules26216482

**Published:** 2021-10-27

**Authors:** Shushan Ge, Jihui Li, Yu Yu, Zhengguo Chen, Yi Yang, Liqing Zhu, Shibiao Sang, Shengming Deng

**Affiliations:** 1 Department of Nuclear Medicine, The First Affiliated Hospital of Soochow University, Suzhou 215006, China; geshushan0820@163.com (S.G.); valdes06@163.com (J.L.); yuyu333222111@126.com (Y.Y.); 2 Nuclear Medicine Laboratory of Mianyang Central Hospital, Mianyang 621099, China; maiwang342@163.com (Z.C.); z531343461@163.com (L.Z.); 3 Department of Nuclear Medicine, The Affiliated Suzhou Science & Technology Town Hospital of Nanjing Medical University, Suzhou 215163, China; yaungyi@163.com; 4 State Key Laboratory of Radiation Medicine and Protection, Soochow University, Suzhou 215123, China

**Keywords:** breast cancer, HER2, radionuclide molecular probes, molecular imaging, clinical trials

## Abstract

As the most frequently occurring cancer worldwide, breast cancer (BC) is the leading cause of cancer-related death in women. The overexpression of HER2 (human epidermal growth factor receptor 2) is found in about 15% of BC patients, and it is often associated with a poor prognosis due to the effect on cell proliferation, migration, invasion, and survival. As a result of the heterogeneity of BC, molecular imaging with HER2 probes can non-invasively, in real time, and quantitatively reflect the expression status of HER2 in tumors. This will provide a new approach for patients to choose treatment options and monitor treatment response. Furthermore, radionuclide molecular imaging has the potential of repetitive measurements, and it can help solve the problem of heterogeneous expression and conversion of HER2 status during disease progression or treatment. Different imaging probes of targeting proteins, such as monoclonal antibodies, antibody fragments, nanobodies, and affibodies, are currently in preclinical and clinical development. Moreover, in recent years, HER2-specific peptides have been widely developed for molecular imaging techniques for HER2-positive cancers. This article summarized different types of molecular probes targeting HER2 used in current clinical applications and the developmental trend of some HER2-specific peptides.

## 1. Introduction

Breast cancer (BC) has surpassed lung cancer as the most frequently occurring cancer worldwide, with 2.3 million new cases diagnosed in 2020 (11.7% of all cancers), and BC ranks the fifth cause of cancer-related death overall and the leading cause of cancer death in women [1]. BC is a highly heterogeneous type of tumor [2], and it can be mainly categorized into four different subtypes: luminal A, luminal B, HER2 (human epidermal growth factor receptor 2)-positive, and triple-negative [3]. Currently, estrogen receptor (ER), progesterone receptor (PR), and HER2 are three main clinical therapeutic targets for BC [2,4,5]. The overexpression of HER2 is found in about 15% of BC patients [6]. Furthermore, the overexpression of HER2 is often associated with a poor prognosis due to the effect on cell proliferation, migration, invasion, and survival [7,8,9].

Targeting HER2 with a range of anti-HER2 drugs has been successfully applied in clinical practice and significantly improved patient outcomes in both advanced and early disease settings [10]. However, there is increasing evidence of temporal and spatial heterogeneity in BC HER2 overexpression [11,12,13]. About 10.3% of patients will have inconsistent HER2 expression between primary breast tumors and distant metastases [13]. Therefore, accurate assessment of HER2 expression plays an essential role in cancer diagnosis and treatment.

The biopsy-based diagnosis has been proved to be clinically useful [11]. However, it is difficult to determine the heterogeneity and status discordance between primary and metastatic tissues using biopsy [14]. Moreover, bone metastases are frequently detected in BC patients, and it is difficult to access their status by using biopsy [15]. Compared with biopsy, molecular imaging with anti-HER2 probes allows the non-invasive, whole-body assessment of tumor burden and may enable the selection of patients for HER2-targeted therapy, dosage optimization, schedule of treatment, and assessment of response to anti-HER2 therapies [16,17,18,19]. This approach can reveal intertumoral or intratumoral heterogeneity as well as variations in HER2 expression over time.

**Figure 1:**
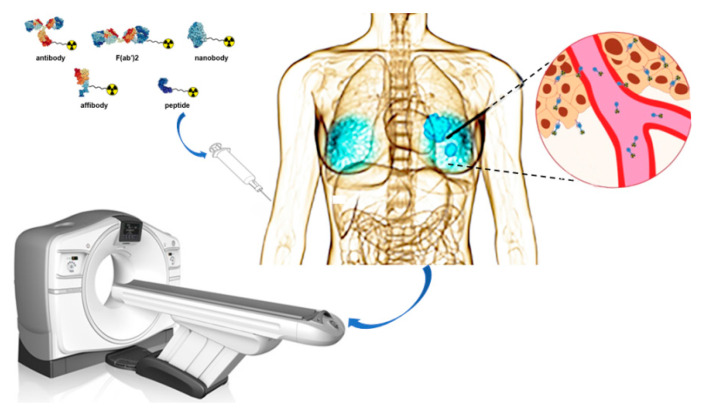
Schematic overview of nuclear imaging for Her2 positive BC. Ligands such as antibody, F(ab’)2 fragments, nanobody, affibody, and peptide can be coupled to a chelator. The chelator enables labeling with different radionuclides that can be applied for imaging purposes.

In the present work, we focused on radionuclide molecular probes targeting HER2 and provided a comprehensive overview of different molecular probes used by the current clinical applications (Figure 1). Moreover, we also summarized the developmental trend of some HER2-specific peptides.

## 2. Radionuclides

Single-photon emission computed tomography (SPECT) and positron emission tomography (PET) are the basic radionuclide imaging modalities [20]. Table 1 lists the commonly used radionuclides for SPECT and PET. The nuclides ^99m^Tc and ^111^In are widely used in SPECT. However, their poor diagnostic performance limits their clinical application. With the increasing popularity of cyclotrons, a variety of novel positron-emitting radionuclides (such as ^18^F, ^64^Cu, ^68^Ga, and ^89^Zr) have been widely used in PET imaging. Compared with SPECT, PET has better sensitivity, spatial resolution, and quantization accuracy [21].

## 3. Radiolabeled HER2-Targeted Monoclonal Antibodies

**Figure 2:**
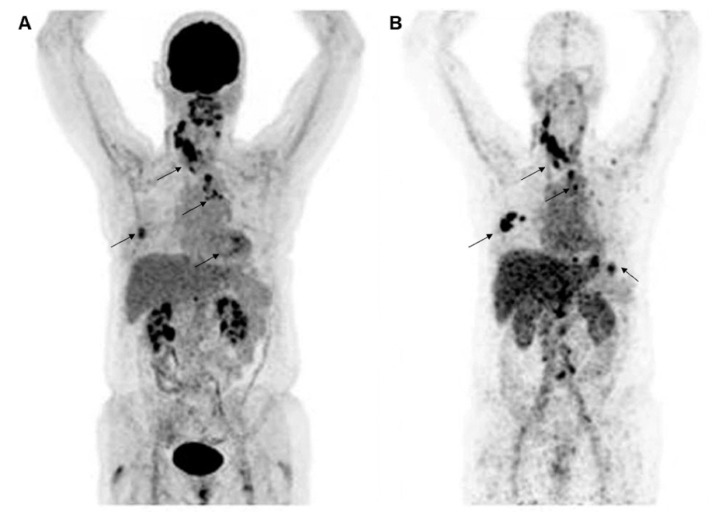
Representative molecular PET images of a patient with HER2-positive BC were visualized by ^18^F-FDG (**A**) and HER2 (**B**) imaging with ^89^Zr-trastuzumab. Images are reproduced with permission from [30].

As the first humanized mAb against HER2, trastuzumab (Herceptin) is approved by the Food and Drug Administration (FDA) for HER2-positive BC, and it has been widely labeled by radionuclide, including ^111^In, ^124^I, ^64^Cu, and ^89^Zr [22,23,24,25]. Of them, two initial clinical studies using ^111^In-DTPA-trastuzumab have reported the therapeutic effect and cardiotoxicity of patients with HER2-positive metastatic breast cancer (MBC) treated with trastuzumab [22,26]. Nonetheless, the poor resolution of SPECT may lead to a lower detection rate of HER2-positive tumors. However, trastuzumab labeled with positron nuclides ^89^Zr and ^64^Cu significantly improves the image quality. Several clinical studies (Table 2) have shown the value of ^89^Zr-Df-trastuzumab in detecting HER2-positive lesions and predicted the response to treatment with trastuzumab (Figure 2) as well as other types of treatment and trastuzumab-related toxicity [27,28,29,30]. However, Ulaner et al. have presented findings from a prospective clinical trial of ^89^Zr-trastuzumab PET/CT in a total of 20 patients with HER2-positive metastases and HER2-negative primary BC [27,28]. Three of these patients develop HER2-positive disease, which is confirmed by immunochemistry (IHC)/fluorescence in situ hybridization (FISH) (NCT02286843). A multi-center study (NCT01565200) consisting of 60 MBC patients reported by Gebhart et al. [31] has investigated the association between the [^89^Zr]-trastuzumab uptake and outcome after treatment with trastuzumab-emtansine (T-DM1). The study has shown that HER2-targeted imaging in combination with early metabolic response assessment is of great significance for judging the heterogeneity of MBC and for selecting patients who would benefit from T-DM1 treatment. Trastuzumab labeled with ^64^Cu is an alternative that has also been tested in clinical practice [24,32]. A first-in-human feasibility study of ^64^Cu-DOTA-trastuzumab has demonstrated successful tumor uptake and visualization of HER2-positive primary BC and metastatic lesions in the brain [24]. The poor visualization of liver metastases due to high background uptake in the liver is a limiting factor for its clinical application. However, if cold trastuzumab is given in advance, high liver uptake can be reduced [33,34].

In addition to trastuzumab, pertuzumab is another FDA-approved mAb targeting HER2. In nuclear medicine, this humanized mAb has been labeled with a variety of radiometals [35,36,37,38,39] and proposed to be used as the imaging agent during monotherapy with trastuzumab [40], since its binding site of HER2 (domain II) is different from trastuzumab (domain IV). The first-in-human trial has demonstrated that ^89^Zr-pertuzumab is a clinically valuable HER2-targeted imaging agent for MBC patients [37]. In addition, a prospective clinical trial (NCT02286843) has demonstrated that ^89^Zr-pertuzumab PET/CT helps identify patients with HER2-positive metastases and HER2-negative primary BC [36].

## 4. Radiolabeled HER2-Targeted Antibody Fragments

The Fab and F(ab′)2 fragments of anti-HER2 antibodies, trastuzumab, and pertuzumab labeled with several different radionuclides have been evaluated for imaging in preclinical models [41,42,43,44]. However, all tested probes have a common feature that HER2-positive tumors can be clearly imaged at 24 h after injection. As reported by Smith-Jones [41], ^111^In-DOTA-(Fab′)2 provides a tumor-to-blood ratio of 10 already at 24 h, and the tumor uptake of [^111^In]-DOTA-(Fab′)2 (~20% ID/g) is much lower compared with [^111^In]-DOTA-trastuzumab (≈50% ID/g). This finding reflects the general feature of antibody fragments, showing lower tumor uptake compared with parental antibodies. Reilly et al. [43] have reported that ^64^Cu-NOTA-F(ab′)2-pertuzumab can detect changes in the expression of HER2 in response to trastuzumab while delivering a lower total body radiation dose compared with ^111^In-labeled pertuzumab. Beylergil et al. [44] have reported a clinical study on the diagnostic utility of ^68^Ga-DOTA-F(ab∲)2-trastuzumab in 15 BC patients (NCT00613847). The results of clinical evaluations are hardly encouraging, since only 4/8 patients with HER2-positive BC are detected.

The long waiting time for imaging of intact radiolabeled antibodies can be solved by using radiolabeled (Fab∲)2 and Fab fragments. However, similar to intact antibodies, the contrast of the main metastatic sites of radiometal-labeled fragments still remains the main problem limiting its clinical application.

## 5. Radiolabeled HER2-Targeted Nanobodies

Due to the small size and high affinity, nanobodies can penetrate tumor tissues and bind antigens with high specificity, making them a suitable therapeutic and diagnostic tool [45] (Table 3). As an sdAb developed against HER2, 2Rs15d has shown excellent targeting of HER2 in both preclinical settings [46] and clinical settings [47]. A phase I study of ^68^Ga-NOTA-2Rs15d reported by Keyaerts et al. [47] has shown that its biodistribution is favorable with a fast blood clearance. The primary lesions of BC patients show a wide uptake of 0.7-11.8, while significant uptake is observed in most metastatic lesions, indicating that ^68^Ga-NOTA-2Rs15d may not be suitable to be used to evaluate the expression of HER2 in primary BC lesions. Currently, a phase II clinical trial (NCT03331601) to evaluate its potential for detecting brain metastases in BC patients and a phase II clinical trial (NCT03924466) to evaluate the correlation between image-based HER2 quantification in the uptake of ^68^Ga-NOTA-2Rs15d in local or distant metastasis in BC patients are all in progress.

What is more, nanobody 2Rs15d is also labeled with various nuclides, such as ^18^F, ^99m^Tc, ^131^I, and ^177^Lu. Similar to ^68^Ga-NOTA-2Rs15d, ^99m^Tc-2Rs15d [48] and [^18^F]RL-II-2Rs15d [49] show high tumor uptake, rapid blood clearance, low accumulation in non-target organs other than the kidneys, and a high tumor to background ratio. Furthermore, the PET imaging of ^18^F-labeled 2Rs15d with different chelators [49,50] shows significant tumor uptake and substantially lower renal uptake.

2Rs15d is also labeled with a therapeutic radionuclide. In the preclinical study, ^131^I-SGMIB-2Rs15d [51] and ^177^Lu-DTPA-2Rs15d [52] have been shown to significantly prolong median survival. A phase I clinical trial (NCT02683083) of ^131^I-SGMIB-2Rs15d in healthy volunteers and patients with HER2-positive BC shows a high tumor to background ratio, rapid blood clearance, and elimination of unbound nanobodies via the kidney after intravenous administration.

In addition, two single-domain antibodies (MM-302 and NM-02) have been labeled and entered clinical studies. The tumor accumulation of ^64^Cu-MM-302 ranges from 0.52 to 18.5 %ID/kg after 24-48 h, including deposition in bone and brain lesions [53]. It is worth noting that significant background absorption of ^64^Cu-MM-302 is observed in the liver and spleen. Currently, the phase I clinical trial of ^64^Cu-MM-302 (NCT02735798) has been terminated. At present, no results have been reported for another ^99m^TC-labeled nanobody NM-02 that has entered the clinical early phase I study (NCT04040686).

## 6. Radiolabeled HER2-Targeted Affibodies

Affibody [54] is a triple helix structure composed of 58 amino acids, which is also called an “artificial antibody”. In contrast to antibodies, the smaller affibody molecules have relatively fast uptake and clearance rates [55]. In addition, the affibody molecules have high affinity and stability, and thus, they are very well suited for molecular imaging (Table 4). The anti-HER2 affibody molecule ZHER2:342 and its derivatives are second-generation HER2-targeted affibodies, which are the most studied affibody imaging probes radiolabeled with different nuclides [56,57,58]. ABY-002 (DOTA ZHER2:342 pep2) is a derivative of ZHER2:342 with a DOTA coupled to its NH2 terminus. The first clinical trial of the radiolabeled HER2-targeted affibody ABY-002 labeled with ^111^In and ^68^Ga has demonstrated their potential for visualizing HER2-expressing metastatic lesions in BC patients [59].

As another HER2-targeted affibody, ABY-025 (ZHER2:2891) has been investigated in clinical trials. The ^111^In-ABY-025 [60] has demonstrated favorable biodistribution, safety, and tumor-targeting potential in patients with HER2-expressing MBC (NCT01216033). It is worth noting that one patient with MBC and HER2-positive primary tumor imaging by ^11^^1^In-ABY-025 shows a HER2-negative status of the metastases, as confirmed by IHC. Two other clinical trials (NCT02095210 and NCT01858116) have studied the effects of two different doses of ^68^Ga-ABY-025 (100 μg or 500 μg) on tumor uptake. The PET image shows that the injection of 500 μg ^68^Ga-ABY-025 leads to a better specificity after 2 to 4 h and allows the differentiation of metastases with HER2 expression levels of 3+ and 2+ [61,62]. ^68^Ga-ABY-025 is currently being investigated in further clinical research. Furthermore, ABY-025 is also labeled with ^18^F by using three methods (such as ^18^F-SiFA, ^18^F-AlF-NOTA, and ^18^F-FBA) [63]. Glaser et al. [64] have found that ^18^F-FBA-ZHER2:2891 (GE226) with enhanced pharmacokinetic characteristics can differentiate tumors with different expression levels of HER2 by imaging. The efficacy of [^18^F]GE-226 in determining the expression of HER2 in MBC patients is being investigated (NCT03827317). No results have yet been reported for this study yet. Another ^99m^Tc-labeled affibody (HPArk2) is also being investigated in an open-label phase I clinical trial, while no results have been published (NCT04267900).

Hober et al. have reported the development of an affibody ADAPT6 that binds HER2 [65]. The pharmacologic studies have demonstrated that ^111^In/^68^Ga-DOTA-(HE)3-ADAPT6 is specifically taken up by HER2+ tumors, with a high tumor-to-normal tissue ratio in mice xenograft tumor models. Furthermore, they have evaluated the effects of the commonly used macrocyclic chelators NOTA, NODAGA, DOTA, and DOTAGA on the biodistribution characteristics of ADAPT6 [66]. It has been concluded that ^111^In-(HE)3DANS-ADAPT6-GSSC-DOTA is best for SPECT imaging, while ^68^Ga-(HE)3DANS-ADAPT6-GSSC-NODAGA is best for PET imaging. The phase I clinical study has shown that ^99m^Tc-ADAPT6 is safe, and a protein dose of 500 μg can be used to distinguish tumors with high or low expression of HER2 as early as 2 h after injection [67]. The above-mentioned results indicate that ADAPT6 is a potential molecular imaging probe.

## 7. Radiolabeled HER2-Targeted Peptides

Peptides have many favorable characteristics suitable for the development of imaging agents. First of all, unlabeled small molecule precursors have the advantages of definite chemical structure, flexible modification space, controllable pharmacokinetic properties, non-immunogenicity, and ease of synthesis commonly involving solid-phase peptide synthesis. Secondly, radionuclide-labeled peptides have higher tissue penetration and faster circulation time in the blood [68,69]. Therefore, imaging of HER2-expressing tumors with radionuclide peptides can lead to quicker imaging time and provide accurate assessment results for treatment based on the expression of HER2. Many peptides have shown promising application value in the detection of HER2 expression in BC (Table 5).

### 7.1. KCCYSL-Based Peptides

One of the most studied peptides specific for HER2 is the KCCYSL peptide or peptides with KCCYSL incorporated into the sequence. The peptide KCCYSL is first found by Karasseva et al. [70] from a random six-amino acid peptide bacteriophage display library and shows a strong affinity to HER2. Then, it has been evaluated in multiple studies both in vitro and in vivo. KCCYSL is radiolabeled with ^111^In and ^64^Cu by Kumar et al. for the imaging of HER2-positive tumors [71,77]. The cell-binding experiments have shown that the radiolabeled peptides ^111^In-DOTA(GSG)-KCCYSL and ^64^Cu-(GSG)-KCCYSL can specifically bind to MDA-MB-435 cells (HER2+), while the scrambled peptide ^111^In-DOTA(GSG)-KYLCSC peptide does not bind to MDA-MB-435 cells. In vivo studies have confirmed that imaging with radiolabeled peptides can specifically identify HER2+ tumors and HER2- in SCID mice at 2 h after injection.

Larimer et al. [72] have developed two peptides, MEGPSKCCYSLALASH (1-D03) and GTKSKCCYSLRRSS (3-G03), which have higher binding affinity compared with the original peptide. Each peptide is radiolabeled with ^111^In for further studies. Among them, the biodistribution of ^111^In-DOTA-1-D03 shows that the ratio of tumor to blood after 2 h is about 6. The SPECT imaging shows that radiolabeled peptides can clearly image HER2+ tumors and show specific binding.

The KCCYSL peptides show great potential as a targeting agent for the development of HER2. Radiolabeled versions of this peptide have been shown to have suitable imaging capabilities with SPECT and are hopefully developed as a PET imaging agent.

### 7.2. LTVSPWY-Based Peptides

Shadidi et al. [73] have used another phage display biopanning procedure that results in the discovery of another HER2-specific peptide (LTVSPWY). Several peptides with LTVSPWY incorporated into the sequence have been radiolabeled by ^99m^Tc and 68Ga for imaging studies of HER2-positive tumors. Sabahnoo et al. [78] have used ^99m^Tc to label two peptides with core LTVSPWY and a cysteine-based ligand (CGGG or CSSS). The ^99m^Tc-CGGG-LTVSPWY and ^99m^Tc-CSSS-LTVSPWY peptides show significantly higher binding to SKOV-3 (HER2+) cells compared with A549 and MCF-7 (HER2-) cells. The competitive binding of trastuzumab has confirmed that these peptides have similar binding sites as trastuzumab. In in vivo studies, mice bearing SKOV-3 xenografts are injected with each peptide to determine the tumor%ID/g after 1 and 4 h. The ^99m^Tc-CGGG-LTVSPWY has a tumor %ID/g of 3.84 ± 2.5 and 2.44 ± 1.1%ID/g at 1 and 4 h, respectively. The ^99m^Tc-CSSS-LTVSPWY peptide has similar values of 4.98 ± 4.8 and 2.26 ± 2.1%ID/g, respectively, at the corresponding time points.

The team of Hosseinimehr has conducted multiple studies on the same core peptide [74,75,79,80]. They have used multiple nuclides to label the peptide with the addition of a new chelator and linker system, SSSLTVPWY. It has been shown that ^99m^Tc-HYNIC-(Ser) 3-LTVPWY can specifically target HER2+ tumors, including SKOV-3 ovarian cancer, at 4 h post-injection, while U-87 MG glioma-based tumors can be visualized after just at 1 h post-injection. In addition, they have also used the EDDA/tricine mixture as a co-ligand to label HYNIC-SSSLTVSPWY with ^99m^Tc. In vivo experiments have shown that ^99m^Tc-HYNIC-(EDDA/tricine)-peptide exhibits noticeable tumor uptake compared with non-target organs after 4 h. However, the ratios of the tumor to blood, or the tumor to muscle for ^99m^Tc-HYNIC-(Ser)3-LTVPWY are 1.32 and 2.65, respectively, and ^99m^Tc-HYNIC(EDDA/tricine)-(Ser)3-LTVSPWY with higher ratios (6.93 and 4.05 respectively) results in better pharmacokinetic modification. The positron nuclide ^68^Ga is also used to label SSSLTVSPWY. The PET imaging experiments exhibit a capacity of ^68^Ga-DOTA-(Ser)3-LTVSPWY for imaging the HER2-positive tumors in nude mice with high contrast.

### 7.3. The Peptide AHNP

The peptide AHNP is obtained by analyzing the CDR3 loop of the heavy chain of trastuzumab that binds to the antigen through a structure-based method by Park et al. [76]. The AHNP has an amino acid sequence of FCGDFYACYMDV with a binding affinity for HER2 of 395 nM. Guan Siao-Syun et al. [81] have used ^111^ In to label AHNP-PEG with the chelator DTPA and performed potential imaging in patients with HER2-positive gastric cancer. In vivo imaging experiments in xenografts mice induced by NCI-N87 have shown that ^111^In-DTPA-AHNP-PEG can clearly identify HER2-positive gastric tumors at 1, 4, 24, and 48 h.

### 7.4. The Peptides H6F and H10F

The H6F(YLFFVFER) [82] and H10F(KLRLEWNR) [83] peptides reported by the group of Wang [84] are discovered by an efficient peptide screening strategy based on in situ single-bead sequencing microarray, and both peptides show high binding and selectivity to HER2. They have used ^99m^Tc to label these two peptides with the chelator HYNIC. ^99m^Tc-HYNIC-H6F and ^99m^Tc-HYNIC-H10F display excellent HER2-binding specificity both in vitro and in vivo. SPECT/CT imaging with ^99m^Tc-HYNIC-H6F/^99m^Tc-HYNIC-H10F in MDA-MB-453, SK-BR3, and MDA-MB-231 xenograft mice model reveals that the HER2+ tumors are clearly visualized, whereas the signals from HER2- tumors are much lower. HER2-positive tumor uptake of ^99m^Tc-HYNIC-H6F and ^99m^Tc-HYNIC-H10F can be blocked by excess unlabeled H6F and H10F but not by excess trastuzumab. In the preliminary clinical study, the ^99m^Tc-HYNIC-H10F has shown potential applications on non-invasive tumor classification, as well as prediction and monitoring of trastuzumab therapeutic efficacy.

Furthermore, Wang et al. [85] have designed a retro-inverso D-peptide of H6 (RDH6) to increase the metabolic stability and added PEGylation to improve its water solubility and in vivo pharmacokinetics. The results show that the D-amino acids ^99m^Tc-PEG4-RDH6 bring better metabolic stability compared with ^99m^Tc-PEG4-H6 with higher tumor uptake. In addition, the introduction of PEG can effectively increase tumor uptake, among which ^99m^Tc-PEG24-RDH6 has a comparable tumor uptake and the lowest liver radioactivity. The SPECT imaging demonstrates that ^99m^Tc-PEG24-RDH6 can specifically distinguish HER2-positive tumors from HER2-negative tumors with better imaging contrast, indicating that the ^99m^Tc-PEG24-RDH6 has the potential for clinical screening of HER2-positive BC.

### 7.5. Other Peptides

Other potential peptides are also promising for the development of HER2 imaging agents. A9 peptide [86] is synthesized based on the design of trastuzumab (Fab) fragment, and it is synthesized with the chelator DTPA for labeling with ^111^In. The BT474 cell-binding experiment has revealed the presence of two ligand-binding sites on the receptor target, one with an affinity of K_d_ = 4.9 nM and the other one with a weaker affinity (K_d_ = 103 nM). This finding shows that more work is warranted for the A9 peptide in future studies.

## 8. Conclusions and Outlook

As a broad tumor biomarker, HER2 plays an important role in tumorigenesis. Therefore, accurate assessment of HER2 expression in cancer patients is essential for cancer diagnosis and treatment. Several different anti-HER2 probes, such as monoclonal antibodies, antibody fragments, Affibody molecules, and nanobodies, developed for both SPECT and PET have successfully reached the clinical setting for assessing whole-body HER2 expression patterns (Table 6). Although mAb molecular probes can identify HER2-positive lesions, slow blood clearance, low sensitivity, terrible tumor specificity, and non-specific uptake are still the main problems that limit its clinical transformation. HER2 imaging based on Fab and (Fab’)2 fragments shows good imaging capabilities within 24 h after injection and can solve the issue of long waiting time between the injection of intact radiolabeled antibodies and imaging. However, it must be noted that the tumor uptake of (Fab’)2 fragment is lower than the peak uptake of the parental antibody. Preclinical and clinical studies of small molecular probes, such as nanobodies and affibodies, have shown that they may be the most promising imaging agents. Their high affinity, specific targeting, and rapid clearance rate make it possible for them to evaluate the intertumoral expression heterogeneity and alteration of HER2 status during the disease. However, they still have the problems of high liver and kidney background, high cost, and may not be suitable for the evaluation of HER2-positive primary lesions.

In recent years, more and more small molecule peptides have been developed for HER2 imaging. The HER2-targeted peptides have shown strong potential for imaging HER2-positive tumors. The benefit for the development of these peptides includes the potentially quicker circulation time, deeper tissue penetration, non-immunogenicity, and ease of preparation. However, low tumor uptake levels and tumor-to-organ ratios are still the main problems to be solved in the development of short radiolabeled peptides. One of the main reasons is that the binding affinity of peptides is generally low. Therefore, more research on radiolabeled peptides is required to promote its clinical transformation.

At present, it is hard to say which type of HER2 tracer will be preferred in the clinical setting. We propose that the practical purpose of HER2 imaging will dictate the choice of imaging molecular probes. For instance, low molecular weight probes can be used as an alternative IHC or FISH to diagnose HER2 expression levels in tumors. In contrast, mAbs may be preferred for evaluating the efficacy of a monoclonal antibody-based therapy (e.g., trastuzumab, T-DM1, pertuzumab).

Based on existing preclinical and clinical studies, we are optimistic that HER2-targeted imaging can provide a non-invasive, dynamic visualization and quantification of HER2 expression status in heterogeneous tumors, which will improve the clinical management of HER2-targeted therapy.

## Figures and Tables

**Table 1:** Nuclides used in PET and SPECT imaging.

Nuclide	T_1/2_	Production Method
Nuclides for SPECT imaging		
^99m^Tc	6.01 h	^99^Mo/^99m^Tc Generator
^123^I	13.3 h	Cyclotron
^111^In	2.8 days	Cyclotron
Nuclides for PET imaging		
^13^N	9.97 min	Cyclotron
^11^C	20.4 min	Cyclotron
^68^Ga	67.6 min	^68^Ge/^68^Ga Generator
^18^F	109.8 min	Cyclotron
^64^Cu	12.7 h	Cyclotron
^89^Zr	78.4 h	Cyclotron
^124^I	100 h	Cyclotron

**Table 2:** The clinical trials of HER2-targeted monoclonal antibodies.

Monoclonal Antibodies	Nuclide/Chelator or Linker	Modality	Condition or Disease	Phase
Trastuzumab	^89^Zr-Df	PET	HER2-Positive Solid Tumor	Phase II NCT04757090
Trastuzumab	^89^Zr	PET	Metastatic Breast Cancer	Phase II NCT01832051
Trastuzumab	^89^Zr-Df	PET/MRI	Breast Cancer	Early Phase I NCT03321045
Trastuzumab	^89^Zr-DFO	PET	Esophagogastric Cancer	NCT02023996
Trastuzumab	^64^Cu-DOTA	PET	HER2 Positive Breast Carcinoma	Phase II NCT02827877
Trastuzumab	^64^Cu	PET	HER2+ Metastatic Breast Cancer	Phase I NCT00605397
Trastuzumab	^111^In-DTPA	SPECT	Breast Cancer Prostate Cancer Lung Cancer Colon Cancer	Early Phase I NCT01445054
Pertuzumab	^89^Zr-DFO	PET	HER2-Positive cancer	Phase I NCT03109977
Pertuzumab	^89^Zr-SS ^89^Zr	PET	HER-2 Positive Malignant Carcinoma of Breast HER2-Positive Metastatic Breast Cancer	Phase I NCT04692831
Pertuzumab	^111^In	SPECT	Breast Cancer	Phase I NCT01805908

**Table 3:** The clinical trials of HER2-targeted nanobodies.

Nanobodies	Nuclide/Chelator or Linker	Modality	Condition or Disease	Phase
2Rs15d	^68^Ga-NOTA	PET	Breast Carcinoma Brain Metastasis of Breast Carcinoma	Phase II NCT03331601, NCT03924466
2Rs15d	^131^I	SPECT	Breast Cancer	Phase I NCT02683083
NM-02	^99m^Tc	SPECT	Breast Cancer	Early Phase I NCT04040686
MM-302	^64^Cu	PET	Advanced HER2+ Cancers with Brain Mets	Early Phase I NCT02735798

**Table 4:** The clinical trials of HER2-targeted affibodies.

Affibodies	Nuclide/Chelator or Linker	Modality	Condition or Disease	Phase
ABY-025	^68^Ga	PET	HER2-Positive Breast Cancer, Breast Cancer	Phase I/II NCT02095210 NCT01858116
ABY-025	^111^In	SPECT	Breast Cancer	Phase I/II NCT01216033
ABH2	^99m^Tc	SPECT	Breast Cancer	Early Phase I NCT03546478
HPark2	^99m^Tc	SPECT	Breast Cancer	Early Phase I NCT04267900
GE-226	^18^F	PET	Breast Cancer	NCT03827317
ADAPT6	^99m^Tc	SPECT	Breast Cancer	NCT03991260

**Table 5:** Specific HER2-targeted peptides.

Peptide	Labeling Strategy	Modality	Kd (nM)	Reference
KCCYSL	^111^In-DOTA-GSG	SPECT	295 ± 56	[64]
MEGPSKCCYSLALASH	^111^In-DOTA	SPECT	236 ± 83	[67]
GTKSKCCYSLRRSS	^111^In-DOTA	SPECT	289 ± 13	[67]
CGGGLTVSPWY	^99m^Tc	SPECT	4.3 ± 0.8	[69]
CSSSLTVSPWY	^99m^Tc	SPECT	33.9 ± 9.7	[69]
SSSLTVPWY	^99m^Tc-HYNIC	SPECT	2.6 ± 0.5	[70]
SSSLTVPWY	^99m^Tc-HYNIC-EDDA/tricine	SPECT	3.3 ± 1.0	[71]
SSSLTVPWY	^68^Ga-DOTA	PET	2.5 ± 0.6	[72]
FCGDFYACYMDV	^111^In-DTPA-peptide-PEG	SPECT	300	[73]
H6F	^99m^Tc-HYNIC	SPECT	7.48 ± 3.26	[74]
H10F	^99m^Tc-HYNIC	SPECT	NA	[75]
A9	^111^In-DTPA	SPECT	4.9/103	[76]

**Table 6:** The advantages and shortcomings of different types of molecular probes.

Molecular Probes	Advantage	Shortcoming
Monoclonal antibodies	Identify HER2-positive lesions	Low blood clearance, low sensitivity, terrible tumor specificity, and non-specific uptake, higher radiation dose
Antibody fragments	Imaging capabilities within 24 h	Low tumor uptake, low lesion detection rate
Nanobodies	Low molecular weight, high stability, nanomole level affinity, better tumor penetration	Higher kidney background, unsuitable for evaluation of her2-positive primary breast lesions
Affibodies	Different binding sites from monoclonal antibodies, picomole level affinity	Higher liver and kidney background
Peptides	Potentially quicker circulation time, deeper tissue penetration, non-immunogenicity, ease of preparation	Low tumor uptake and tumor-to-organ ratios

## Data Availability

Not Applicable.

## Sample Availability

Samples of the compounds are not available from the authors.
